# Reasons given by women for discontinuing the use of progestogen implants at Koster Hospital, North West province

**DOI:** 10.4102/safp.v64i1.5471

**Published:** 2022-11-02

**Authors:** Bolarinwa T. Olaifa, Henry I. Okonta, Justin B. Mpinda, Indiran Govender

**Affiliations:** 1 Department of Family Medicine and Primary Health Care, Faculty of Health Sciences, Sefako Makgatho Health Sciences University, Pretoria, South Africa

**Keywords:** Implanon, progestogen, implants, Implanon, discontinuing, side effects

## Abstract

**Background:**

In 2014, the South African National Department of Health introduced a new addition to the long-acting reversible contraceptive (LARC) options available in the country. This was a single rod subdermal progestogen implant (Implanon^®^NXT) which provided 3 years of effective contraception cover. However, the new contraceptive device uptake and general acceptance amongst women quickly diminished, with a slew of requests for its removal. The aim of this study was to explore the reasons given by women for discontinuing the use of their progestogen implants at Koster Hospital, North West province, South Africa.

**Methods:**

A qualitative study was conducted using semistructured interviews. Thirteen women were purposively selected and interviewed at Koster Hospital Family Planning Unit. The transcriptions of the audio-taped interviews were analysed thematically.

**Results:**

The following themes emerged from the interviews as reasons the women discontinued their progestogen implants: side effects such as menstrual problems, arm discomfort and weight gain. Other themes were family or social factors and the desire to conceive.

**Conclusion:**

The reasons for discontinuation of Implanon by women at Koster Hospital were the undesirable side effects they experienced whilst using the contraceptive device. These side effects were mainly menstrual problems, arm discomfort and weight gain. Family and other social dynamics also influenced some of the participants’ decision to discontinue their contraceptive implants.

## Introduction

According to a recent report from the World Health Organization (WHO), over 200 million women of child-bearing age in low-income and middle-income countries do not have access to modern family planning methods. This global unmet need for contraception is caused by various reasons, such as limited choice of methods, limited access to contraceptives (especially amongst teenagers), the fear of side effects and finally because of gender, cultural and religious opposition. All these limit the use of modern contraceptives with subsequent consequences.^1^ The use of modern contraceptive methods, such as oral contraceptive pills, injectables and implants, to name a few, has increased in many parts of the world, especially in Asia and Latin America, but continues to be low in sub-Saharan Africa. Globally, the use of modern contraception has risen slightly, from 54.0% in 1990 to 57.4% in 2015. In different regions of the world, contraceptive use amongst women of reproductive age has risen minimally or plateaued between 2008 and 2015. In Africa it went from 23.6% to 28.5%, in Asia it has risen slightly from 60.9% to 61.8%, and in Latin America and the Caribbean it has remained stable at 66.7%.^2^ It has also been observed that globally 6% of women using modern contraceptives use injectables and 1% use implants. Injectables are the predominant contraceptive used in sub-Saharan Africa, accounting for 43.0% of modern contraceptives.^3^

Progestin-only contraceptive implants are cheap, easily reversible and long-acting. They offer highly proficient reversible contraception with sterilisation-like efficacy.^4^ The contraceptive implant has also been listed by the United Nations Commission on Life-Saving Commodities for Women and Children as one of its 13 Life-Saving Commodities.^5^ The South African contraception experience over the years has been dominated by oral hormonal contraceptives, progestogen-only injectable methods and permanent methods such as tubal ligation.^6^ In this vein, the National Department of Health introduced a new addition to the long-acting reversible contraceptives (LARCs) methods in the public health sector, the single rod subdermal progestogen implant (Implanon^®^NXT), which provides three years of effective contraception cover. Other LARCs include intrauterine devices and progestogen depot injectables. The LARC methods have the unique advantage of being highly effective, not relying on compliance and correct use by the client and being reversible. Despite these advantages, they tend to be underutilised in many countries where they have been in use.^7^

The contraceptive method was introduced in South Africa in 2014, and it was designed to give contraceptive effect for an average of three years. Women, for various reasons, are now discontinuing the implant after a couple of months, despite it being a very effective LARC method. The researcher also noted at Koster Hospital that the majority of women who had the Implanon^®^ NXT removed after the three years did not want to have it reinserted because of unpleasant side effect experiences. Recent figures from the district health barometer (2015–2016) show that the introduced progestogen implants contributed 3.6%, versus 7.6% from the previous year (2014–2015), to the total contraceptive years dispensed.^8^ Therefore, the aim of this study was to explore the reasons given by women for discontinuing the use of their progestogen implants at Koster Hospital, North West province, South Africa.

## Methods

A qualitative study using a phenomenological approach, with the aid of in-depth one-on-one interviews, was conducted at the Family Planning Unit of Koster Hospital. The hospital has 50 beds, and it is situated in Koster in the Kgetlengrivier local municipality of the North West province, Republic of South Africa. The Family Planning Unit runs weekdays and sees an average of 200 patients per month. Services include provision of modern contraceptives, termination of pregnancy and Pap smears. There are three clinics (Reagile, Swartruggens and Derby) in its catchment area.

A purposive sampling method was used to select women who had come to remove their progestogen implants at Koster Hospital Reproductive Unit from 01 November 2016 to 30 December 2016. Semistructured in-depth interviews were conducted with 13 participants, as data saturation was achieved whilst interviewing the 12th and 13th participants.

Rapport was established, making the participants feel comfortable. Participants were informed about the purpose of the study, and they gave written consent to take part in the study and for audio-recordings of the interviewed data. Those who could not speak English had their interviews conducted in Setswana using a translated version of the semistructured interview guide. The same open-ended exploratory question was asked to all participants, after which they were given enough time to narrate their experience: ‘Can you tell me what influenced your decision to remove the implants?’, which was asked in English and Setstwana (see Appendix 1).

All interviews were audio-recorded, and a research diary was kept of all the events taking place during the study. The interviews took between 20 and 30 min to complete. In the end, eight interviews were conducted in English, whilst five interviews were conducted in Setswana. The participants were not required to provide any personal information and all personal information was kept secure.

### Data analysis

Data analysis entailed confirming responses for participants and reviewing responses that either were missed or needed a more detailed description. The content of the data was deductively analysed manually after the tapes were transcribed verbatim, and the transcriptions were checked against the tapes for any omissions and inaccuracies.

The interview guide was translated from English to Setswana by a reputable professional translation specialist, reflecting the meaning of the original document. The authenticity of the translation of the five Setswana interviews and their responses was further confirmed by the research assistant and a participant who understood both languages by first scoping out the text to be translated. This was followed by an initial translation. This was further reviewed for accuracy, and finally, the final edits were made to refine the translated text to Setswana. Thereafter, a back-translation from Setswana to English was done to see if the back-translated version retained the information or meaning in the original English version that was translated into Setswana. The outcome was a satisfactory translation, which further ensured the quality of the data.

The research diary and each transcript were reread several times by the researcher, during which recurrent themes were noted. A code chart was prepared for line-by-line open coding, and the codes were assigned meanings. The researcher also involved an independent coder in qualitative research to review the research diary and transcripts and analyse the codes. The two researchers discussed until agreement was reached on the final codes.

### Trustworthiness

Credibility was achieved by prolonged engagement with participants and member checking.^9^ This enabled them to volunteer more sensitive information, rather than preferred social responses. Follow-up interviews were conducted for 11 of the participants for respondent verification. The remaining two follow-up interviews were done in person because the participants did not have cell phones and were contacted via their next of kin.

The researcher gave a thick description, that is, a detailed description, of the research methods. This included the study setting and the selection of participants done by purposive sampling to enhance transferability.^10^ The dependability of this study was achieved by ensuring that the research methods used were described step-by-step in sufficient detail for easy replication by other researchers.^11^ Furthermore, additional notes were made in a research diary. Triangulation of data sources was done by keeping a detailed record of the transcribed tape records and field notes from respondent verification.^11^ Finally, the interviewing researcher (B.T.O.) established confirmability via mechanisation by ensuring that all interviews were audio-recorded, typed and stored electronically. B.T.O. also ensured reflexive analysis was done by keeping a field journal, outlining his personal attitudes and feelings about the study so that they were not imposed on the participants. Further member checks were done after transcription by giving the participants feedback on the interpretations and conclusions reached on the information they provided during the interviews.

### Ethical considerations

Ethical clearance was obtained from the Sefako Makgatho Health Sciences University Research Ethics Committee and North West Department of Health Research Committee (reference number: SMUREC/M/225/2016: PG). Permission to conduct the study was obtained from the management of the Bojanala Subdistrict and North West Department of Health Research Committee.

## Results

A total of 13 participants were interviewed in this study. The demographic characteristics of the participants are presented in Table 1. The participants in the study were denoted as P1–P13 according to the sequence of conducting the interviews.

**TABLE 1:** Profile of study participants.

Participant	Age (year)	Marital status	Number of children	Previous contraceptive use	Employment status
P1	26	Single	1	Injectable	Unemployed
P2	18	Single	0	None	Unemployed
P3	24	Single	2	Injectable	Unemployed
P4	32	Single	5	None	Employed
P5	30	Married	2	Injectable	Employed
P6	34	Married	3	OCP	Employed
P7	38	Married	4	Injectable	Unemployed
P8	23	Married	1	OCP	Unemployed
P9	18	Single	0	None	Unemployed
P10	31	Single	2	IUCD	Employed
P11	26	Married	1	Injectable	Employed
P12	20	Single	0	None	Unemployed
P13	33	Single	3	Injectable	Unemployed

OCP, oral contraceptive pills; IUCD, intrauterine contraceptive device.

Three main themes and seven subthemes emerged from the interviews and are presented in Table 2 with supporting quotes. Most participants cited only one reason to discontinue the implant except two of the participants, P7 and P10, who cited more than one.

**TABLE 2:** Themes and subthemes for discontinuation of implant.

Themes	Subthemes	Supporting quotes from participants
1. Undesirable side effects	1.1 Menstrual problems	
	1.1.1 Heavy periods	‘I had to remove the implant because my periods became very heavy and I was losing a lot of blood.’ (P1, female, 26 years old)
	1.1.2 Continuous periods or spotting	‘I decided to remove the implant because I was bleeding continuously every day since I had it on.’ (P13, female, 33 years old)
	1.2 Arm discomfort	
	1.2.1 Pain at insertion site	‘I decided to remove the implant because there was this pain that did not go away in the arm where it was inserted.’ (P5, female, 30 years old)
	1.2.2 Arm numbness	‘I was feeling weakness and numbness in my arm where the implant was inserted.’ (P4, female, 32 years old)
	1.3 Weight gain	
	1.3.1 Excessive weight gain after insertion	‘I noticed that I was gaining too much weight after the implant was inserted.’ (P6, female, 34 years old)
		‘I decided to remove the implant because I was spotting and putting on weight.’ (P7, female, 38 years old)
2. Family or social factors	2.1 Partner disapproval of contraceptive choice	‘My husband told me to remove it after he found out that I won’t have another baby for three years.’ (P8, female, 23 years old)
	2.2 Discouragement from community members	‘I decided to remove the implant because my neighbour said I might struggle to get pregnant later, that the implant is for older women who have completed childbirth.’ (P9, female, 18 years old)
	2.3 Reduced sexual activity related to bleeding issues	‘I am removing the implant because my partner and I are no longer having sex like before because of the bleeding, which is not stopping.’ (P10, female, 31 years old)
3. Desire to conceive	3.1 Changed mind and desired pregnancy	‘I got engaged shortly after I inserted the implant and now I want to get pregnant and have a baby.’ (P11, female, 26 years old)

**FIGURE 1:**
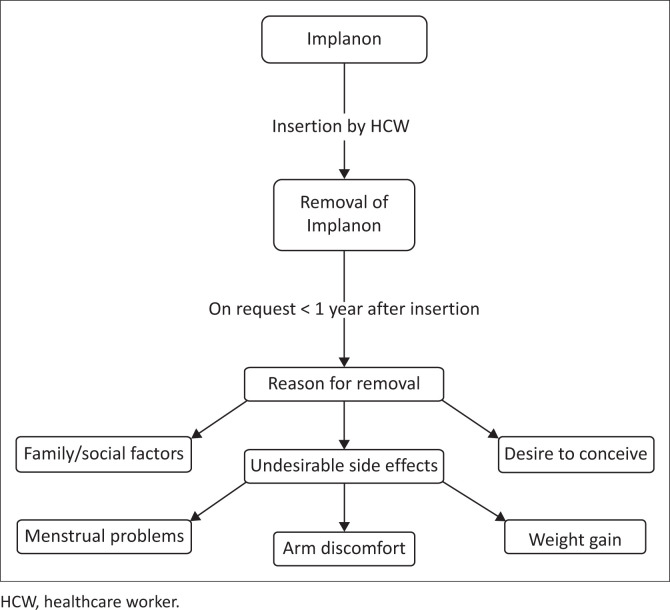
Integration of themes.

The integration of themes or how they relate to one another is shown in Figure 1.

## Discussion

The reasons for discontinuing the progestogen implant that emerged from this study included side effects, namely, menstrual problems, arm discomfort and weight gain. Other reasons included family or social problems and the desire to conceive. Some women had more than one reason to discontinue the implant.

### Theme 1.1: Menstrual problems

In this study, the majority of participants expressed their dissatisfaction with changes in menstrual bleeding, predominantly in the form of prolonged bleeding and spotting. This constituted the main or partial reason for the discontinuation of the implant. Similar results were found in an Australian qualitative study exploring the experiences of women using the subdermal implant. The study found that bleeding-related issues were the main undesirable experience that led to the earlier than expected discontinuation of the implant in the Australian women.^12^ This finding is consistent with the results of Mansour et al. in their integrative analysis of bleeding patterns associated with Implanon, in which 11.3% of women discontinued their contraceptive implants because of unpleasant changes in their menstrual bleeding pattern, especially prolonged flow and frequent irregular bleeding.^13^ It is in line with the results of Jacobson et al., where changes in menstrual bleeding were the major cause for contraceptive discontinuation.^3^ It is also similar to the findings of an Ethiopian study by Alemayehu et al., in which 30.0% of the participants had severe irregular bleeding attributable to low prevalence of contraceptive implant reinsertion.^14^

However, in a West African study on the safety and efficacy of Implanon, 31% of participants expressed dissatisfaction with bleeding irregularities. Despite this, the overwhelming outcome of the study was favourable to the inherent qualities of LARC, such as convenience, long duration of action and low risk of pregnancy. The study culminated in a 93.8% continuation rate after a 12-month period.^15^ This indicates that bothersome side effects such as the bleeding irregularities can be effectively managed and anticipated by adequately informing and counselling women prior to insertion and managing bleeding promptly with appropriate medication.^16^

### Theme 1.2: Arm discomfort

In this study, a couple of participants complained of neuropathic symptoms after Implanon insertion, and this was manifested in the form of motor weakness in the left arm and forearm in one participant, whilst the other complained of persistent pain localised to the nondominant arm where the implant was inserted as recommended by the manufacturer. These findings are similar to the acute ulnar nerve neuropathy in a patient, post insertion of a contraceptive implant, as documented in a case report from the United Kingdom.^17^ It is also in line with another case report in which a young woman presented with forearm pain and hypoesthesia associated with the insertion of the Implanon contraceptive implant.^18^

According to a United States Food and Drug Administration report on Implanon, the fundamental basis of successful use and subsequent removal of the implant is dependent on proper subdermal implant insertion. This can be demonstrated by ensuring that the woman and health care professional can both palpate the implant immediately post insertion and during subsequent check-up visits.^19^ Nevertheless, in extremely rare cases, incorrect placement of the implant, especially deeper insertions, can result in more than nerve injury. There have been reports of migration from the insertion site into the vasculature, with the implant ending up in the pulmonary artery.^20^

### Theme 1.3: Weight gain

A few of the participants in this study expressed their displeasure with weight gain as the main or partial reason for the implant removal. The weight gained by participants was determined by self-weighing at home. A majority of women are very conscious about their weight for both aesthetic reasons and health reasons.^21^ With the current worldwide obesity epidemic and its attendant morbidity, this is a particular side effect of Implanon that should not be taken lightly.^22^ The study findings are in line with the results of several other studies on early removal of Implanon because of weight gain. In an Australian retrospective chart audit that accessed the continuation rates and reason for removal of Implanon amongst users, Harvey found that weight gain was often cited as a reason for removing the implant.^23^ Likewise, in another Australian qualitative study, Flore found that weight gain was one of the main factors identified by participants for having the Implanon devices removed prior to the 3-year expiration period.^24^ In an Ethiopian study, Birhane also found that weight gain was one of the common reasons for discontinuation of Implanon.^25^

### Theme 2: Family or social factors

Two women in this study discontinued the implant because of influences that directly impacted their relationship with their male partners, whilst another participant discontinued the implant because of misinformation from a community member. The effect of gender dynamics in a heterosexual relationship cannot be underestimated, especially in a patriarchal society like South Africa. For instance, traditional gender norms are widely adhered to and the man is considered the head of the home who makes the decisions in a relationship. Therefore, it will be expedient for women in committed relationships to tailor their contraceptive choices to match their partner’s fertility intentions, compared to single women who engage in more casual relationships.^26^ This was evident in participant (P8), who had the implant inserted without her partner’s consent, only to be mandated to discontinue it because of her partner’s disapproval on the basis of it being at odds with his fertility intentions for their relationship. This scenario is consistent with the dismal levels of spousal communication about fertility and family planning in Africa.^27^ Similarly, in an Angolan study, a frequently cited reason for contraceptive implant discontinuation was partner dissatisfaction. This was mainly because of the lack of partner authorisation and conflicting fertility goals between couples, with male partners desiring more children. Some women were even subjected to domestic violence because of it.^28^

At the time of this study, Implanon was still relatively new in the public health sector. This may have given impetus to the misinformation peddled to one of the participants that resulted in the untimely removal of the implant. This view was further supported by a Nigerian study which made significant complementary associations; that is, myths and misinformation were key predictors that discouraged the use of modern contraceptive methods.^29^ Likewise in Ethiopia, a study revealed myths and misconceptions attributed to a negative attitude towards the use of long-acting permanent contraceptives (LAPM), which include intrauterine devices, hormonal implants, female sterilisation and vasectomy. Women who did not subscribe to myths or misconceptions were 1.7 times more likely to be favourably disposed to use the LAPM.^30^ In the United States (US), misconception in adolescent patients requiring contraceptives affected their choices negatively and was considered as one of the access barriers for LARC as a first-line contraceptive choice in this age group.^31^

### Theme 3: Desire to conceive

The essence of modern contraceptive methods is to empower women to plan and space their families. Women are also enabled to determine when they want to get pregnant and the number of children they intend to conceive. Even though a woman is well within her right to insert and remove the Implanon at any time she chooses, a participant (P11) decided to remove the implant in order to get pregnant after getting engaged to her partner some months after the insertion. This removal of a contraceptive implant in a bid to conceive is consistent with results from other studies. In a study of Angolan women, Qui found the desire to conceive to be a key factor for contraceptive implant discontinuation.^28^ In another study by Casner, 3% of participants requested removal of their contraceptive implants within six months of insertion because of their desire to conceive.^32^ Higgins also found in his study that early discontinuation of contraceptive implants was premised on the intention to conceive.^33^

Lastly, some of the participants provided more than one reason for the removal of the implant. In other words, the untimely decision to remove the implant involved the weighted pressure of more than a single undesirable side effect and other factors. These culminated in significant physical and emotional distress to the overall well-being of the participants. This phenomenon was appropriately characterised as the objectives of an Australian study aimed at describing women’s nuanced responses and characterising their multidimensional and complex reasons for discontinuing the use of the contraceptive implant.^12^ A similar multidimensional and complex reason for discontinuing the use of contraceptive implants was observed by Casey in her study of reasons for removal of etonogestrel implant in the US.^34^ Burke et al. also had a similar finding of multidimensional reasons for the discontinuation of injectable contraceptives in their study of Kenyan women.^35^

A strength of this study was its ability to explore the lived experiences of women on Implanon who decided to discontinue it prematurely in a South African setting. A major limitation of this study was the lack of diversity amongst the participants, as all of them were black African women living in the Koster area. Hence, the results may not be reflective of the broader South African demographic. Similarly, the participants were limited to those who could communicate to researchers in either English or Setstwana.

The following measures are recommended to address the reasons why women discontinue progestogen implants. Firstly, guidelines and protocols should incorporate adequate counselling prior to insertion of Implanon, including topics such as side effects of Implanon implant, short-term and long-term fertility goals, relationship status and possible influences of romantic partners on their contraceptive choices. Secondly, it should explore steps to manage anticipated common side effects associated with this method of contraception to avoid future early discontinuations. Further research is recommended to investigate the effectiveness of interventions, such as adequate counselling prior to insertion and prompt management of side effects, on the discontinuation rates of women with contraceptive implants.

## Conclusion

The reasons for discontinuation of Implanon by women at Koster Hospital were the undesirable side effects they experienced whilst using the contraceptive device. These side effects were mainly menstrual problems, arm discomfort and weight gain. Family and other social dynamics also influenced some of the participants’ decision to discontinue their contraceptive implants.

## Appendix 1: Interview guide

**Participants’ number or initials**

### Section A: Basic demographic information

1. Age
2. Relationship status
3. Number of children
4. Previous type of contraceptive use
5. Employment status

### Section B: Modified interview schedule template

**Establish rapport (shake hands):** I am Dr B.T. Olaifa, a family medicine registrar, currently interested in exploring the perspectives of women who discontinue progestogen implants at Koster Hospital, North West province.

Permit me to ask you some questions about why you decided to discontinue the progestogen implant and what influenced your decision.

This information will be utilised to educate the health care professionals to understand better the perspectives of women who decide to discontinue the progestogen implants. It will also help in formulating appropriate counselling strategies and patient education prior to the insertion of the progestogen implants.

The interview may take about 30 min, depending on your answers. Are you available to respond to some questions at this time?

Be informed that all information gathered will be kept confidential.

- Can you tell me what influenced you to remove the implant?

Prompts:

- Why did you decide to remove the implant?
- What information were you given before inserting the implant?

The researcher will use clarifying questions in order to extract more information from the participant when necessary.

Thank the patient for their time and cooperation.
